# Different Distribution Patterns between Putative Ercoid Mycorrhizal and Other Fungal Assemblages in Roots of *Rhododendron decorum* in the Southwest of China

**DOI:** 10.1371/journal.pone.0049867

**Published:** 2012-11-21

**Authors:** Lifu Sun, Kequan Pei, Fang Wang, Qiong Ding, Yanhong Bing, Bo Gao, Yu Zheng, Yu Liang, Keping Ma

**Affiliations:** 1 State Key Laboratory of Vegetation and Environmental Change, Institute of Botany, Chinese Academy of Sciences, Beijing, China; 2 College of Life Sciences, Shaoxing University, Shaoxing, China; University of Illinois at Chicago, United States of America

## Abstract

Fungal diversity within plant roots is affected by several factors such as dispersal limitation, habitat filtering, and plant host preference. Given the differences in life style between symbiotic and non-symbiotic fungi, the main factors affecting these two groups of fungi may be different. We assessed the diversity of root associated fungi of *Rhododendron decorum* using internal transcribed spacer (**ITS**) sequencing and terminal restriction fragment length polymorphism (**T-RFLP**) analysis, and our aim was to evaluate the role of different factors in structuring ericoid mycorrhizal (**ERM**) and non-ericoid mycorrhizal (**NEM**) fungal communities. Thirty-five fungal operational taxonomic units (**OTU**s) were found in roots of *R. decorum,* of which 25 were putative ERM fungal species. Of the two main groups of known ERM, helotialean fungi were more abundant and common than sebacinalean species. Geographic and host patterning of the fungal assemblages were different for ERM and NEM. The distribution of putative ERM fungal terminal restriction fragments (TRFs) showed that there were more common species within ERM than in the NEM fungal assemblages. Results of Mantel tests indicated that the composition of NEM fungal assemblages correlated with geographic parameters while ERM fungal assemblages lacked a significant geographic pattern and instead were correlated with host genotype. Redundancy analysis (**RDA**) showed that the NEM fungal assemblages were significantly correlated with latitude, longitude, elevation, mean annual precipitation (MAP), and axis 2 of a host-genetic principle component analysis (PCA), while ERM fungal assemblages correlated only with latitude and axis 1 of the host-genetic PCA. We conclude that ERM and NEM assemblages are affected by different factors, with the host genetic composition more important for ERM and geographic factors more important for NEM assemblages. Our results contribute to understanding the roles of dispersal limitation, abiotic factors and biotic interactions in structuring fungal communities in plant roots.

## Introduction

Ericaceae is a large, globally distributed family of woody plants [1] that are especially common in low nutrient sites at high latitude or high elevation. Plants belonging to the Ericaceae usually have a unique mycorrhizal association, called ericoid mycorrhiza (ERM) that is thought to be essential for their growth and survival in nutrient-poor and stressful environments where they thrive [2]. ERM are characterized by the presence of “hair roots” with swollen uniseriate cells that are colonized internally by mycorrhizal fungi [3].

Ericaceous plants were once thought to form ericoid mycorrhizal associations with a relatively narrow range of ascomycetous fungi [2], [4]–[6]. However, recent studies suggested that a broader range of ascomycetous fungi and some basidiomycetous fungi can form associations with roots of ericaceous plants [7]–[12]. Furthermore, Ericaceous plants can also be colonized by fungal endophytes, pathogens, or saprophytes.

Fungal diversity in plant roots may depend on geographic dispersal of fungal species, habitat filtering by abiotic factors (e.g. temperature, precipitation, edaphic parameters), and selection by the host plant. Since spores and mycelial fragments of fungal species are small in size, their dispersal rate is potentially high. Consequently, fungal species have been considered cosmopolitan in their distribution according to the hypothesis of Wilkinson [14]. The results of many recent studies, however, have shown that these microorganisms may also have biogeographical patterns similar to higher plants and animals (e.g. [15]–[17]). Because of dispersal limitation of fungal species and spatial auto-correlation of environmental factors, dissimilarities between fungal assemblages in soils of study sites are expected to increase with geographic distance. Fungal diversity in plant roots is also determined by specificity or preference of plant-fungi associations [18],[19]. Fungal species lacking host preferences would be expected to be selected randomly from soils and their occurrences would depend much on geographic factors. Fungal species with high host preferences, such as mycorrhizal fungi and some endophytic fungi, are expected to be strongly structured by host genetics. The distribution of fungal species with high host preferences may be independent of geographic factors if dispersal is not limited and if the hosts themselves are not geographically patterned.

Our goal was to study the geographic and host patterns for ERM and non-ericoid non-mycorrhizal (**NEM**) fungi associated with the roots of a widespread ericaceous plant, and to examine the roles of geographic, host genetic and other abiotic factors on these two distinct categories of fungi. We chose *Rhododendron decorum* (Ericaceae) as our study plant because it is a widespread evergreen shrub or small tree, native to Sichuan, Yunnan, Xizang, and Guizhou provinces in the southwest of China [13]. *R*. *decorum* is found in the sub-alpine zone from 1000 to 3300 m in altitude, and has been introduced to many arboretums and botanic gardens all over the world due to its beautiful flowers. *R*. *decorum* is a typical ERM plant species and its individuals form ericoid mycorrhizal structures - hair roots - with diverse ERM fungi [12]. Thus, in addition to answering questions about the roles of geography versus plant hosts in structuring fungal communities, studies on fungal diversity of *R*. *decorum* growing in sites with different climatic and edaphic conditions can help determine the factors driving the structure of root-associated fungal communities.

We measured the diversity and composition of *R*. *decorum* root-associated fungal communities and the genetic similarities among multiple *R*. *decorum* individuals. We used these results to (1) determine the geographic patterns of ERM and NEM fungal assemblages in roots of *R*. *decorum*, and (2) evaluate the role of geographic distance, soil abiotic factors, and host genetic similarity in structuring ERM and NEM fungal assemblages.

## Results

### 2.1 Climatic, Edaphic and Plant Element Parameters of Sampling Regions

**Figure 1:**
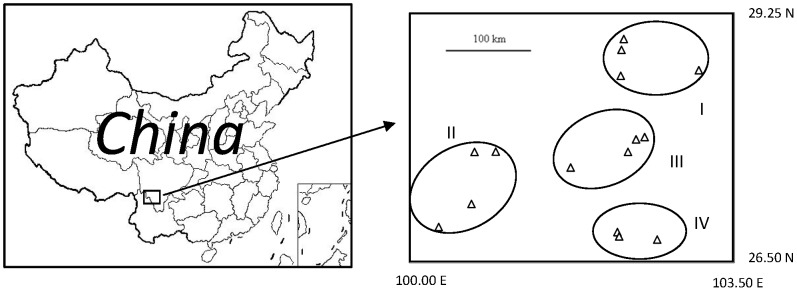
Sampling regions and study sites of the present research.

Climatic, edaphic and plant element parameters of four sampling regions (see Fig. 1) are shown in Table S1. Mean annual temperature (MAT) was highest in region IV and lowest in region I, and mean annual precipitation (MAP) was highest in region IV and lowest in region II. Soil total C (STC) and total P (STP) showed no significant differences between regions, while soil total N (STN) was significantly higher in region IV than in regions II and III. Soil pH in region IV was significantly lower than those of other regions. Plant leaf total C in region II was significantly lower than those in other regions. Leaf total N was highest in region IV. Leaf total P in region IV was significantly higher than that in region I.

### 2.2 Genetic Structure of R. decorum Population

Using five inter-simple sequence repeat (**ISSR**) primers, 119 clear and reproducible ISSR bands were obtained from 45 *R. decorum* individuals. Sørensen similarity indices between plant individuals were from 0.356 to 0.835.

**Figure 2:**
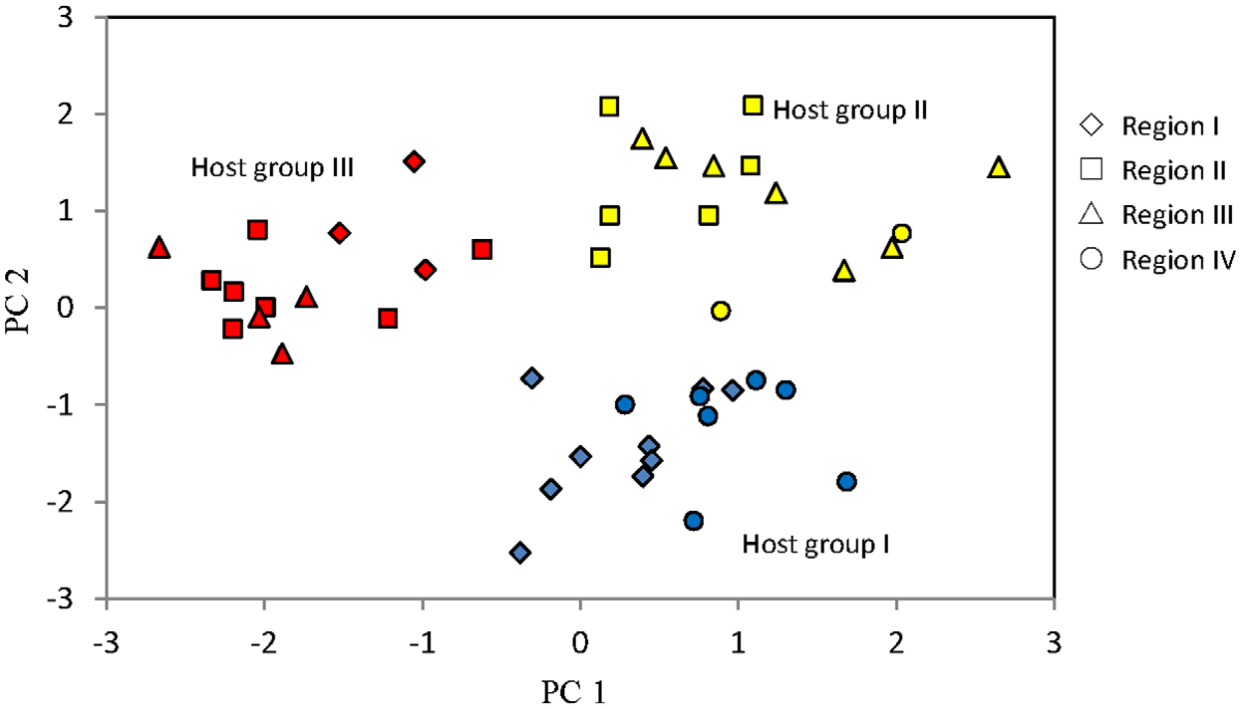
Principle component analysis (PCA) based on genetic relations of *R. decorum.* * R. decorum* individuals from four regions (shown in different shapes) were separated into three groups (shown in different colors).

A principle component analysis (**PCA**) analysis of the ISSR data showed that the host population could be divided into three host groups (Fig. 2). Host group I contains individuals from region I and region IV. Host groups II and III include mainly individuals from regions II and III. Host group III separates from the other two groups along the axis of PC1, and host group I separates from other groups along the axis of PC2.

### 2.3 Fungal Diversity in Roots of R. decorum

A total of 75 internal transcribed spacer (ITS) sequences were obtained from roots of *R. decorum* and were clustered into 35 fungal OTUs using a 97% similarity threshold (Table 1). These OTUs include 25 putative ERM fungal taxa, two putative ectomycorrhizal (ECM) fungal taxa, two dark septate endophytic species (DSE) and six uncharacterized taxa. Most of the putative ERM fungal OTUs belonged to the orders Sebacinales (basidiomycetes) and Helotiales (ascomycetes). Using T-RFLP method, 30 out of 35 OTUs were detected in roots of *R. decorum*, including 22 putative ERM OTUs and eight NEM OTUs. On average, 12.2±0.8 fungal OTUs were detected in the roots of each plant. A species accumulation curve showed the relationship between percentage of fungal terminal restriction fragments (TRFs) and number of host individuals sampled (Fig. S1). Sampling and analysis of 20 host individuals were required to obtain 80% of NEM TRFs obtained in the full data set (45 plants), while only five individuals were needed to obtain 80% of ERM TRFs.

**Table 1:** Fungal species associated with roots of *Rhododendron decorum.*

Name	Accession Number	Closest BLAST match	Identity
**Putative ericoid mycorrhizal (ERM) fungi** *			
Sebacinales sp1	HQ850084	Uncultured Sebacinales clone [EU625939.1]	656/714(91%)
Sebacinales sp2	HQ850091	Uncultured Sebacinales clone [FN663852.1]	403/433(93%)
Sebacinales sp3	HQ850092	Uncultured Sebacinales clone [EU625939.1]	663/708(93%)
Sebacinales sp4	HQ850096	Salal root associated fungus [AF284137.2]	414/434(95%)
Sebacinales sp5	HQ850098	Uncultured Sebacinales clone [EF127237.2]	674/693(97%)
Sebacinales sp6	HQ850107	Uncultured Sebacinales clone [FJ552823.1]	645/685(94%)
Sebacinales sp7	HQ850112	Uncultured Sebacinales clone [EF127237.2]	430/438(98%)
Sebacinales sp8	HQ850116	Uncultured Sebacinales clone [EF127237.2]	668/692(96%)
Sebacinales sp9	HQ850117	Uncultured Sebacinales clone [EF127237.2]	681/691(98%)
Sebacinales sp10	HQ850124	Uncultured Sebacinales clone [HQ211970.1]	673/705 (95%)
Sebacinales sp11	HQ850143	Uncultured Sebacinales clone [EF127237.2]	660/692(95%)
Sebacinales sp12	HQ850145	Uncultured Sebacinales clone [EF127237.2]	680/689(99%)
Helotiales sp1	HQ850097	Uncultured Helotiaceae clone [FJ553302.1]	541/579(93%)
Helotiales sp2	HQ850105	uncultured Helotiales [FN565272.1]	533/600 (89%)
Helotiales sp3	HQ850113	uncultured Helotiales [FN565272.1]	569/594 (96%)
Helotiales sp4	HQ850123	Uncultured Helotiaceae clone [FJ553302.1]	540/579(93%)
Helotiales sp5	HQ850135	*Holwaya mucida* [DQ257357.1]	538/592(90%)
Helotiales sp6	HQ850136	Uncultured Varicosporium clone [HQ211582.1]	585/591 (99%)
Helotiales sp7	HQ850137	Uncultured Helotiales clone [GU998282.1]	572/612(93%)
Helotiales sp8	HQ850139	Uncultured Helotiales clone [GU998282.1]	532/573(93%)
Helotiales sp9	HQ850141	Uncultured Helotiales clone [HQ260175.1]	556/597 (93%)
*Oidiodendron maius*	HQ850129	*Oidiodendron maius* isolate [HQ608115.1]	563/582(97%)
*Rhizoscyphus ericae* aggregate sp1	HQ850095	*Rhizoscyphus ericae* aggregate [AM084704.1]	511/579(88%)
*Articulospora tetracladia*	HQ850138	*Articulospora tetracladia* [EU998923.1]	588/590(99%)
Mycorrhizal ascomycete of *Rhododendron*	HQ850090	Mycorrhizal ascomycete of *Rhododendron* type 3 [AB089662.1]	329/347(94%)
**Putative ectomycorrhizal fungi**			
Thelephoraceae sp1	HQ850125	Uncultured Thelephoraceae clone [EF619796.1]	613/680(90%)
Agaricomycete sp1	HQ850144	Uncultured Agaricomycetes clone [FJ553957.1]	620/708(88%)
**Dark septate endophyte**			
*Phialocephala fortinii*	HQ850099	*Phialocephala fortinii* [AY394921.1]	599/605(99%)
Dark septate endophyte 1	HQ850085	Dark septate endophyte [AF168783.1]	661/668(98%)
**Other fungal species with unknown ecological niche**			
Ascomycete sp1	HQ850100	*Neoscytalidium dimidiatum* [FM211431.1]	427/521 (82%)
Ascomycete sp2	HQ850101	Uncultured Ascomycota clone [HM239917.1]	391/467(84%)
Ascomycete sp3	HQ850106	Uncultured Ascomycota clone [HM239716.1]	410/418 (98%)
Ascomycete sp4	HQ850130	Uncultured ascomycete clone [EU490040.1]	373/377(99%)
Pezizomycotina sp1	HQ850140	Uncultured Pezizomycotina clone [FJ554411.1]	565/576 (98%)
Geoglossaceae sp1	HQ850142	*Geoglossum umbratile* [EU784257.1]	800/891(90%)

* OTUs of putative ericoid mycorrhizal (ERM) fungi were considered as ERM fungi in further analyses.

### 2.4 NEM and ERM Fungal Assemblages along Geographic Distances

**Figure 3:**
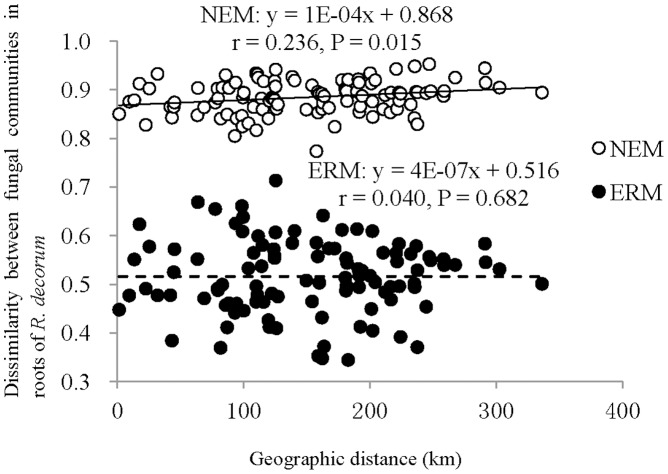
Relationship between geographic distance and dissimilarity of NEM (open circle) and ERM (closed circle) fungal communities in roots of *R. decorum.*

The relationship between geographic distance and dissimilarity of NEM and putative ERM fungal assemblages in roots of *R. decorum* are shown in Fig. 3. ERM fungal assemblages were more similar than NEM fungi at all geographic distances analyzed. A modest, but significant, positive correlation was found between geographic distance and dissimilarity of NEM fungal assemblages in roots of *R. decorum* (*P = *0.015). No significant correlation was found between geographic distance and ERM fungal assemblages. If all fungal TRFs were considered, no significant correlation was found between geographic distance and dissimilarity of fungal assemblages (*r* = 0.093, *P* = 0.346).

### 2.5 Distribution of ERM and NEM Fungi

**Figure 4:**
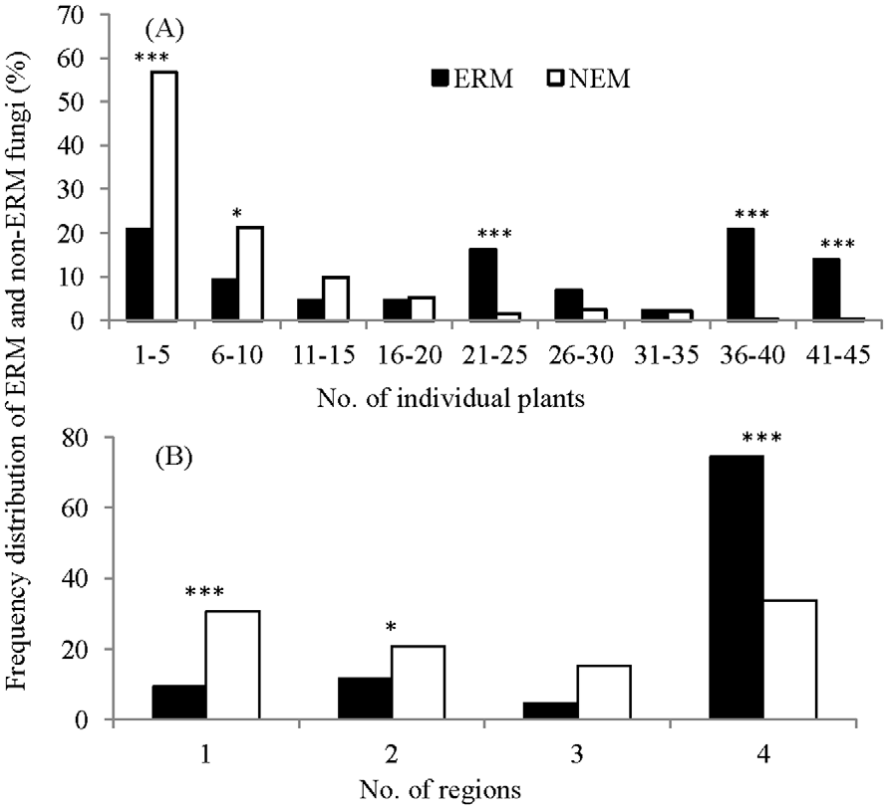
Frequency distribution of TRFs of putative ericoid mycorrhizal (ERM, black) and other (NEM, white) fungi detected in different numbers of *R. decorum* individuals (A) or different numbers of geographic regions (B). * and *** indicate significant differences between proportions of ERM and NEM fungi at P<0.05 and P<0.001 levels.

The frequency distribution of NEM and ERM fungi in different number of host individuals or regions is shown in Fig. 4. The distribution of NEM fungal TRFs shows that most OTUs had relatively low frequency, as most (56.8%) TRFs were detected in the roots of five or fewer individuals. In contrast, the proportion of ERM TRFs with low frequency, those occurring in 5 individuals or less, accounted for only about 20% and this proportion was significantly lower than NEM fungi (Fisher’s exact test, P<0.001). Compared with NEM fungi, ERM TRFs tended to occur in many more host individuals. Over 50% of ERM fungal TRFs were found in groups of 21–25, 36–40, and 41–45 host individuals (Fig. 4A). An analysis of the geographic distribution of NEM and ERM fungi showed a similar pattern of low frequency in NEM versus high frequency in ERM as 51.2% of NEM fungal TRFs occurred in only one or two regions while 74.4% ERM fungal TRFs were found in all the four study regions (Fig. 4B). The results of Fisher’s exact test showed significant differences between NEM and ERM fungi in the proportion of TRFs occurring in 1, 2, and 4 regions (Fig.4).

**Figure 5:**
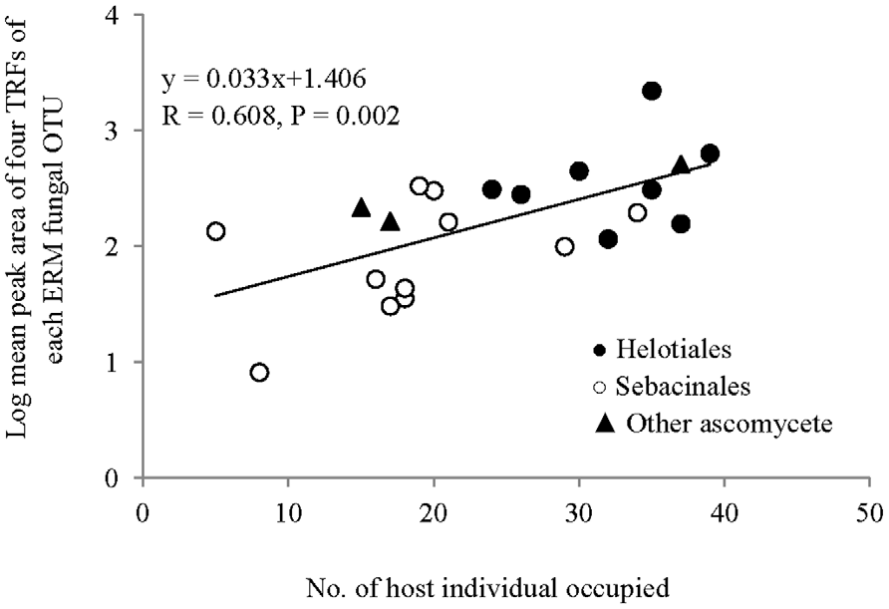
Relationship between abundance of ERM fungal OTU and number of host individual occupied. The abundance of each ERM fungal OTU was calculated as the log of the mean peak area of four TRFs.

The relationship between abundance of putative ERM fungal OTU and the number of host individuals occupied is shown in Fig. 5. A significant positive correlation was found between abundances of putative ERM fungal OTUs estimated by mean peak area of four TRFs and number of host individuals they occupied (P = 0.002). Fungi from the orders Sebacinales and Helotiales were detected at different levels and frequencies in host roots. Helotialean fungi were more abundant and common in roots of *R. decorum* than sebacinalean species (Fig. 5). Results of a cluster analysis of the host distribution patterns of ERM fungal OTUs showed that fungal taxa from the orders Sebacinales and Helotiales had different occurrence patterns on host individuals of different host groups and sampling regions (Fig. S2).

### 2.6 Factors Affecting ERM and NEM Fungal Assemblages

The results of Mantel tests demonstrated that the effect of geographic position, host population genetic structure, climatic matrix (mean annual temperature and mean annual precipitation), leaf and soil nutrient levels on putative ERM and residual NEM fungal assemblages in roots of *R. decorum* (Table 2). The composition of the NEM fungal assemblage in roots of *R. decorum* correlated significantly with geographic distance while ERM fungal assemblages correlated significantly only with host genetic similarity. Neither NEM nor ERM assemblages correlated with climatic matrix and soil or leaf nutrient levels.

**Table 2:** Mantel test results showing effects of geographic position, host population genetic structure, climatic matrix, leaf and soil element composition on putative ericoid mycorrhizal (ERM) and residual (NEM) fungal terminal restriction fragments (TRFs) assemblages in roots of *R. decorum*.

	Geographic position		Host genetic structure		Climatic matrix		leaf C/N/P of host		soil C/N/P	
	*r*	*P*	*r*	*P*	*r*	*P*	*r*	*P*	*r*	*P*
ERM Fungal assemblages	0.029	0.317	**0.174**	**0.045**	−0.031	0.342	−0.069	0.256	−0.028	0.430
NEM Fungal assemblages	**0.101**	**0.033**	0.021	0.361	0.020	0.393	−0.070	0.231	−0.064	0.259

Standardized Mantel statistic (r) values and *P-value* of Monte Carlo test (4999 randomized runs) are shown.

Redundancy analysis (**RDA**) of NEM and ERM fungal assemblages and host populations demonstrated that the factors correlated with host populations were largely different from those correlated with their associated fungal assemblages (Table 3). NEM fungal assemblages were found to correlate with geographic, climatic, edaphic and host genetic factors. Specifically, correlations were found with latitude, longitude, elevation, mean annual precipitation (MAP), and axis 2 of host-genetic PCA (HSTPC2 from Fig 2). In contrast, ERM fungal assemblages only correlated with latitude and axis 1 of host-genetic PCA (**HSTPC1**, PC1 from Fig 2). The genetic structure of host population was correlated with elevation, mean annual temperature (**MAT**), and soil element compositions (soil total carbon (**STC**), soil total nitrogen (**STN**), soil total phosphorus (**STP**), and with axis 2 of host-genetic PCA (**HSTPC2**, PC2 from Fig 2). Of the plant-related factors, only elevation and axis 2 of host-genetic PCA were correlated with fungal assemblages.

**Table 3:** Redundancy analysis (RDA) results showing the influence of geographic, climatic, edaphic and host genetic factors on structure of putative ERM assemblages, residual fungal assemblages and the plant host population.

	ERM community			NEM community				Host population		
	*r^2^*	*P*	*Post-hoc*	*r^2^*	*P*	*Post-hoc*		*r^2^*	*P*	*Post-hoc*
Latitude	0.233	0.004	*	0.586	<0.001		***	0.035	0.468	
Longitude	0.028	0.546		0.442	<0.001		***	0.103	0.104	
Elevation	0.152	0.031		0.283	0.001		**	0.209	0.009	*
MAT	0.095	0.12		0.115	0.075			0.192	0.01	*
MAP	0.106	0.096		0.591	<0.001		***	0.053	0.315	
STC	0.006	0.877		0.066	0.242			0.248	0.003	*
STN	0.024	0.597		0.168	0.02			0.417	<0.001	***
STP	0.183	0.014		0.064	0.249			0.491	<0.001	***
pH	0.171	0.021		0.172	0.02			0.209	0.009	
HSTPC1	0.227	0.004	*	0.179	0.017			1	<0.001	***
HSTPC2	0.129	0.054		0.281	0.001		**	1	<0.001	***

*R^2^* values were calculated using envfit function in the vegan package (R 2.12), and *P* values were based on 9999 permutations.

Post-hoc marked by *, **, *** indicate significances at P<0.05, P<0.01, and P<0.001 after Holm-Bonferroni correction. MAT and MAP are mean annual temperature and mean annual precipitation; STC, STN, and STP indicate soil total carbon, soil total nitrogen and soil total phosphorus; HSTPC1 and HSTPC2 indicate the first two axes of Principle component analysis (PCA) of host population.

## Discussion

### 3.1 Geographic Pattern and other Factors Related to NEM and ERM Fungal Assemblages

While many factors, including plant nutrient status, light intensity, and plant community composition, have been suggested to influence the colonization of mycorrhizal fungi and root endophytes (e.g. [10], [20]–[31]), most of these studies focused on arbuscular mycorrhizal and ECM fungi. Factors that influence or are simply correlated with ERM fungal diversity are still unknown. Different fungal species may have different geographical distributions due to differences in their optimal soil moisture, temperature, etc. For species obligately associated with plants, their distribution pattern must also be limited by the geographical distribution of host plants. Our results showed that NEM fungi in roots of *R. decorum* had obvious geographic patterning. Specifically, increased geographic distance correlated with increased dissimilarity of NEM fungal assemblages. Dispersal limitation is one possible obvious explanation for this correlation, but the distances here are not great (300 km or less) and ERM fungi, which might be expected to have similar dispersal abilities, showed no similar distance decay relationship. Another possibility is that this pattern is driven at least in part by the relative rarity of most NEM fungi (Fig. 4), coupled with limited sampling number. Given that sampling is never complete in these microbial systems, rare species may pass for endemics because the chance of sampling them in a given site is low. In contrast, the ERM species form relatively simple, predicable assemblages relative to the host units that were the focus of our sampling, and therefore were better sampled within our study and less prone to artifactual endemism. In any case, to determine the underlying cause of the geographic correlation seen within the NEM fungi, the identities of these fungi will need to be determined and a sampling scheme targeted toward them will be necessary.

The RDA results showed that among the three geographic factors (latitude, longitude and elevation), all the three factors were related to the structuring of NEM fungal assemblages, while only latitude was related to ERM fungal assemblages. For climatic factors, mean annual precipitation (MAP) was related to NEM fungal assemblages, which indicates that NEM fungal compositions may be more related to precipitation levels at sites. Interestingly, PC1 and PC2 of host-genetic analysis showed significant correlations with ERM and NEM fungal assemblages, respectively, suggesting that different host genetic factors are involved in structuring these two fungal assemblages.

### 3.2 The Role of Intra-species Host Preference in Structuring Mycorrhizal Fungal Community

Our results showed that host genetic structure rather than geographic separation was correlated with ERM fungal assemblages, which suggests that intra-species host preferences play an important role in determining structure of mycorrhizal communities. The mantel test results showed that ERM fungi are affected by host genetic structure (Table 2). Although the plants were grouped into three clusters based on host genetic structure, host genetic structure itself was not strongly associated with geographic parameters (Table 3). Thus, ERM fungi are not strongly affected by geographic separation. Previous studies have shown the importance of the host in structuring ectomycorrhizal (e.g. [17],[18]) and arbuscular mycorrhizal communities (e.g. [32]–[35]), but to our knowledge this is the first study to show such a pattern in ericoid mycorrhizal fungi, and this is also the first to show this pattern at the genetic level within a single species. We did not expect to find the strong pattern of host genetic structure revealed by our data (i.e., Fig 2), and the underlying reasons for it remain unknown. In any case, the striking correlation with ERM fungal assemblages and the lack of correlation with geographic structure, or with soil or plant nutrients, showed that the host plant has a strong impact on ERM assemblages.

### 3.3 Fungal Diversity in Roots of R. decorum

Our results showed that species of Helotiales were common in hair roots of *R. decorum.* In a recent study on culturable ERM fungi of *R. decorum*, Tian *et al*. also found that the four most common ERM fungi belong to Helotiales [12]. Twelve fungal OTUs found in the present study belonged to the order Sebacinales. These organisms also have been found in studies of Ericaceae in roots of *Gaultheria shallon* and *Epacris pulchella* [10].

Some recent studies have suggested that diverse fungi can form associations with roots of ericaceous plants (e.g. [7]–[10]). Our results have shown that some species of DSE, ECM fungi and species without a known ecological niche colonized roots of *R. decorum*, which indicated that *R. decorum* plants can serve as hosts to a diverse range of fungal species.

### 3.4 Distribution of Different ERM Fungal OTUs

Three ecological models – the Brown, Levin and Hanski models – have been used to explain species regional distribution patterns ([36]–[38]). The Brown and Levin models predict unimodal regional distributions while that of Hanski predicts bimodal distributions of species [39].” The “core-satellite” hypothesis, derived from Hanski’s model, defines “core” species as those that are locally abundant and regionally common species and “satellite” species as those that locally and regionally rare [40]. Our results indicated that NEM fungi had a typical unimodal distribution with most TRFs occurring on few host individuals. The frequency distribution of ERM fungi, however, was bimodal as predicted by Hanski's model; this is consistent with the idea that there are more “core” species in the ERM than in the NEM fungal assemblages in roots of *R. decorum.* An important trait of Hanski’s model is the positive correlation between average abundance and number of plots occupied [39], which was also found in the ERM fungal assemblages of our study (Fig. 5). Our results showed that the abundance, as measured by average peak area of ERM fungal OTUs, increased with the increasing of the number of colonized host individuals (Fig. 5).

While ERM fungi can form some specific structures (e.g. hyphal coils in epidermal cells of hair roots) with host plants, it can be difficult to identify ERM fungi in plant roots and to distinguish ERM fungi from other fungi in root fungal communities. In our study, we considered fungal species of Sebacinales and Helotiales in ericoid mycorrhizal roots (hair roots) as ERM fungi. It should be noted that some species of the two fungal orders may be not ERM though they colonized ERM roots [12]. In addition, there are most likely other, as yet unknown, ERM fungi and these have not been identified as such in this study. Including more ERM species in the global ERM fungal molecular databases and the development of new approaches, e.g. fluorescent *in situ* hybridization studies of fungi on these plant roots will be helpful to separate ERM and NEM communities.

## Methods

### 4.1 Study Site

*R. decorum* plants were sampled from fifteen sites from four regions within Sichuan and Yunan provinces (Fig.1). The mean annual temperature of this area ranges from 10.9 to 17.0°C and the mean annual precipitation is from 801 to 1079 mm (based on climate data from Weather China (http://www.weather.com.cn)). The soil types in this area are classified as yellow or red soils (Haplic Acrisols or Haplic Alisols, according to FAO soil classification) in the Chinese soil classification system [41], and soil pH of the study sites is from 4.5 to 8. Common plant species in the sampling area include *Alnus cremastogyne, Pinus armandii, P. densata, Rosa omeiensis, Quercus semicarpifolia, Lithocarpus glabra*, and several other Ericaceous species, e.g. *Rhododendron irroratum, R. spinuliferum, R. racemosum, R. yunnanensis* (Table S2).

### 4.2 Sampling Procedure

Leaves and hair roots of three individuals of *R. decorum* over 20 years old were sampled from each site. In total, 45 leaf and root samples were collected (3 samples × 15 sites). Leaves taken from each host individual were used for host genetic and leaf element analyses. Leaves used for host genetic analyses were kept at −70°C before DNA extraction. The terminal portion of the finer roots (typical ericaceous “hair roots”) was retrieved from soil at three positions around the trunk of *R. decorum* individuals and cleaned carefully after 1 h soaking in tap water. Hair roots were then cut into 1 cm segments and 20 hair root segments were sampled randomly from each *R. decorum* individual. Each root segment was put into a centrifuge tube and preserved in 70% alcohol at −70°C before DNA extraction. A 200 g soil sample for elemental analyses was taken from the top 10 cm of soil adjacent to each plant sampled.

Additional leaf samples of *R. decorum* to be used for elemental analyses were collected as above and oven dried at 70°C for 48 h. These leaves and soil samples used for elemental analyses were ground to pass through an 80-mesh sieve using a mechanical mill (Retsch MM 400, Retsch GmbH & Co KG, Haan, Germany). Total C content was determined using a H_2_SO_4_–K_2_Cr_2_O_7_ oxidation method [42]. Subsamples were digested in H_2_SO_4_–H_2_O_2_ [43] and subjected to total N and total P determinations. Total N content was analyzed with an Alpkem autoanalyzer (Kjektec System 1026 Distilling Unit, Sweden). Total P of the digest was measured colorimetrically at 880 nm after reaction with molybdenum blue [44].

### 4.3 DNA Extraction from Roots and Leaves of R. decorum

DNA was extracted from hair roots and leaves of *R. decorum* following the protocol of Guo *et al*. [45] with slight modifications. One 1 cm hair root segment was put into a sterile centrifuge tube containing 20 µl 2×CTAB extraction buffer solution (2% CTAB, 100 mM Tris-HCl pH 8.0, 20 mM EDTA pH 8.0, 1.4M NaCl), and ground with a plastic pestle on ice. A 630 µl aliquot of 2×CTAB extraction buffer solution was added into the centrifuge tube. Tubes were warmed to 65°C for 1 h, and then shaken for 10 min. A 630 µl aliquot of chloroform/isoamyl alcohol (24∶1) was added, mixed gently and centrifuged at 13,201×g for 8 min at room temperature. The supernatant was transferred into a new centrifuge tube and an equal volume of chloroform/isoamylol (24∶1) was added. After centrifuging at 13,201×g for 8 min, the supernatant was transferred into a new centrifuge tube and DNA was precipitated by adding two volumes of 100% alcohol at 4°C for 1h. After centrifuging at 17968×g for 8 min, the supernatant was discarded, the DNA precipitate was washed twice using 70% ethanol, dried in a vacuum desiccator, and dissolved in 30 µl sterile ddH_2_O at 4°C. DNA sample from each root segment were stored at −20°C prior to downstream analyses.

### 4.4 Mycorrhizal Fungi ITS Amplification and T-RFLP Analysis

To generate sufficient DNA for community analyses, a nested PCR approach was utilized to amplify fragments of fungal ITS region. Primers NSI1 (5′-GATTGAATGGCTTAGTGAGG-3′) and NLB4 (5′-GGATTCTCACCCTCTATGAC-3′) were initially used [46]. The PCR reaction volume was 50 µl, containing 2.0 mM MgCl_2_, 0.2 mM of each dNTP (TaKaRa, Japan), 0.2 µM of each primer, 25 mg BSA, 2 U *Taq* polymerase (TaKaRa, Japan) and 1 µl template DNA (*ca.* 20 ng ). PCRs were performed using a PTC-200 thermocycler (MJ Research, Inc., Watertown, Massachusetts, USA) with an initial denaturation step of 95°C for 8 min followed by 35 cycles of 95°C for 30 s, 60°C for 40 s and 72°C for 40 s, followed by a final extension of 72°C for 5 min. Initially, genomic DNA extracted from 20 root samples from each *R. decorum* individual was used as template for independent PCRs using the primers NSI1 and NLB4. Subsequently, the amplicons from these 20 reactions were pooled as template DNA for the second round PCR amplification. The second round PCR primers were ITS1F (5′-CTTGGTCATTTAGAGGAAGTAA-3′) and ITS4 (5′-TCCTCCGCTTATTGATATGC-3′) [47], labeled with the fluorescent dyes 6-FAM (6-carboxyfluorescein, succinimidyl ester) and HEX (6-carboxy-hexachlorofluorescein, succinimidyl ester) respectively. The second amplifications were performed in 50 µl reaction volumes containing 1.5 mM MgCl_2_, 0.2 mM each dNTP (TaKaRa, Japan), 0.1 µM each primer, 1.5U *Taq* polymerase (TaKaRa, Japan), and 1 µl of the first amplification pooled products. Amplifications were performed using a PTC-200 thermocycler with an initial denaturation step of 94°C for 5 min, 30 cycles of 94°C for 40 s, 55°C for 40 s and 72°C for 40 s, followed by a final extension of 72°C for 10 min. PCR products were electrophoresed on 2% agarose gels, stained with ethidium bromide and visualized under UV light to verify the presence of appropriately sized amplification products.

We constructed an ERM fungal clone library for each host individual using pooled amplicons generated using the nested PCR approach described above. The PCR products were cloned into the pEASY-T1 vector after purification using an Easypure PCR Purification Kit (TransGen Biotech, Beijing, China), according to manufacturer’s instructions. Clones were screened by PCR using the primers ITS1/ITS4F, and by RFLP using three restriction enzymes *Hinf I*, *Hae III* and *Rsa I*. For each unique RFLP profile, three clones were sequenced with a 3730 DNA Capillary Sequencer (Applied Biosystems, USA) by Sunbiotech Co. Ltd. (Beijing, China). A total of 74 sequences were recovered.

We used the software package MOTHUR [48] for trimming, screening, and alignment of sequences, and for distance calculation and assigning sequences to operational taxonomic units (OTU). In this procedure, the nearest neighbor method was used for distance calculations, with 97% similarity as the OTU threshold. Representative sequences from each OTU were queried by BLAST searches against NCBI (National Center for Biotechnology Information) databases [49]. The sequences reported in this paper have been deposited in the GenBank database (accession nos. HQ850084 to HQ850145).

After purification using Easypure PCR Purification Kit, PCR products were digested by two restriction enzymes, *HaeIII* and *HinfI* (TaKaRa, Japan), respectively. The digested products were purified again, and the terminal restriction fragments (TRFs) were separated on an automated sequencer (ABI 3700). Fragment sizes and peak heights were determined with the software package GeneMapper (Life Technologies). Fragments short than 50 bases were ignored in further analyses.

### 4.5 ISSR-PCR Amplification and Electrophoresis

We used Inter-simple sequence repeats (**ISSR**) to determine the genetic variation across plant hosts. Simple sequence repeats **(SSR**, also known as microsatellites**)** are repeating sequences of 2–6 bp of DNA. ISSR are located in genomic regions between microsatellite loci. ISSR can differentiate closely related individuals and has been applied to population-level and interspecific studies of natural populations of plants and animals [50]. Five primers which produced clear and reproducible ISSR bands were selected after an initial screening in a preliminary experiment and synthesized by Shenggong Inc. (Shanghai, China) (Table S3). ISSR amplification reactions were carried out in a volume of 25 µl containing 1.5 mmol/L MgCl_2_, 1×*Taq* DNA polymerase buffer, 0.2 mM each dNTPs (TaKaRa, Japan), 0.2 µM each primer, 1 U Taq polymerase (TaKaRa, Japan), and about 30 ng of template DNA. Negative controls without DNA were also analyzed. PCR amplifications were performed in a PTC-200 thermocycler with an initial denaturation step of 5 min at 94°C, 35 cycles of 94°C for 30 s, 57°C for 60 s, 72°C for 90 s, and a 5 minute final extension at 72°C. The amplification products were screened by electrophoresis on 1.5% agarose gels stained with ethidium bromide. The software package Labworks 4.0 was used for analyzing electrophoresis banding patterns, and a binary ISSR matrix of *R. decorum* individuals was generated based on the presence or absence of ISSR bands of different sizes, ranging from 100 to 3000 bp.

### 4.6 Statistical Analysis

The presence or absence of each fungal member detected in the clone library was determined by searching for the fragments determined by *in silico* digests in the TRFLP profiles from environmental samples. OTU were determined to be present in a root sample if at least three of the four expected peaks for any given sequence (two restriction enzymes, and forward and reverse primer TRFs from each) were detected in digests from that sample (±1 base in size).

The ERM fungal community including putative ERM TRFs as defined in this study is shown in Table 2. After determining the presence/absence of putative ERM fungi from local fungal library, TRFs that were not attributed to ERM fungi were defined as the NEM fungal community. Sørensen dissimilarity was calculated for each of four matrices (one matrix for each terminal end in each of two digests) containing ERM or NEM fungal TRFs. The mean Sørensen dissimilarity index of the four TRF matrices were considered as the community dissimilarity of ERM or NEM fungi between two root samples. In order to test the relationship between geographic distance and dissimilarity of fungal communities in roots of *R. decorum*, Pearson correlation coefficients between geographic distance and dissimilarity of NEM and ERM fungal communities in roots of *R. decorum* were calculated. Differences in the frequency distribution of NEM and ERM fungi by number of host individuals colonized or regions occupied were evaluated using Fisher’s exact test [51].

Genetic similarity between *R. decorum* individuals was estimated using the Sørensen similarity index calculated based on ISSA analyses. To study the distribution-abundance relationship of ERM fungi, regression analyses between abundance of ERM fungal OTU and number of host individuals occupied were carried out (SPSS for windows, ver. 16.0). The abundance of each ERM fungal OTU was estimated by mean peak area of four TRFs and log transformed before regression analysis. Cluster analysis was carried out to show the distribution patterns of fungal OTUs in different host groups and sampling regions using PC-ORD 5.0 [52], distance measurement was based on Bray-Curtis distance and the group linkage method was Nearest Neighbor.

Redundancy analysis (RDA) was conducted using the vegan package of R 2.12 [53] to assess the influence of geographic, climatic, edaphic and host genetic factors on structure of putative ERM TRF assemblages, residual fungal assemblages and host population. *R^2^* values were calculated using the envfit function in the vegan package, and *P* values were based on 9999 permutations. The Holm-Bonferroni correction was used to adjust the P-values of envfit function [54], and the adjusted P-values for Holm–Bonferroni method are: 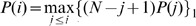, where {x}_1_ ≡ min(x,1), P(j) is the unadjusted P-values listed from lowest to highest and N is the total number of factors tested in RDA. The Mantel test was carried out to show effects of geographic position, host population genetic structure, climatic matrix (MAT and MAP), leaf and soil element composition on putative ERM and NEM fungal TRF assemblages in roots of *R. decorum*, and P-values were calculated by Monte Carlo test (4999 randomized runs). For quantitative matrices, *i.e.* climatic matrix, and leaf and soil element compositions, parameters were relativized to the maximum values of the 45 samples before dissimilarity calculations were performed using Euclidean distances and Mantel test.

### Ethics Statement

No specific permits were required for the described field studies. The study sites are not privately-owned or protected in any way, and the field studies did not involve endangered or protected species.

## Supporting Information

Figure S1 **Relationship between percentage of fungal terminal restriction fragments (TRFs) and number of host individuals sampled. Bars were standard deviations (SD).**
(TIFF)

Figure S2 **Cluster analysis of distribution patterns of individual fungal species based on Bray-Curtis distance of fungal presence/absence data from 45 sampled plants.** The mean relative abundance of each fungal taxon in the three host groups and four sampling regions is shown as a heat-map.
(TIFF)

Table S1 **Climatic, edaphic, and plant element parameters in four sampling regions.** Data were shown MAT and MAP are mean annual temperature and mean annual precipitation; STC, STN, and STP indicate soil total carbon, soil total nitrogen and soil total phosphorus; PTC, PTN, and PTP indicate leaf total carbon, leaf total nitrogen and leaf total phosphorus of host plant. Different letters denote significant differences among regions.
(DOCX)

Table S2 **Sampling sites of** ***Rhododendron decorum.***
(DOCX)

Table S3 **Primers used in ISSR analysis for** ***R. decorum.***
(DOCX)
