# Supplementary figures and images for: Different Distribution Patterns between Putative Ercoid Mycorrhizal and Other Fungal Assemblages in Roots of Rhododendron decorum in the Southwest of China
Source: PLoS One. 2012 Nov 21;7(11):e49867. doi: 10.1371/journal.pone.0049867 (PMC3504031; doi:10.1371/journal.pone.0049867)

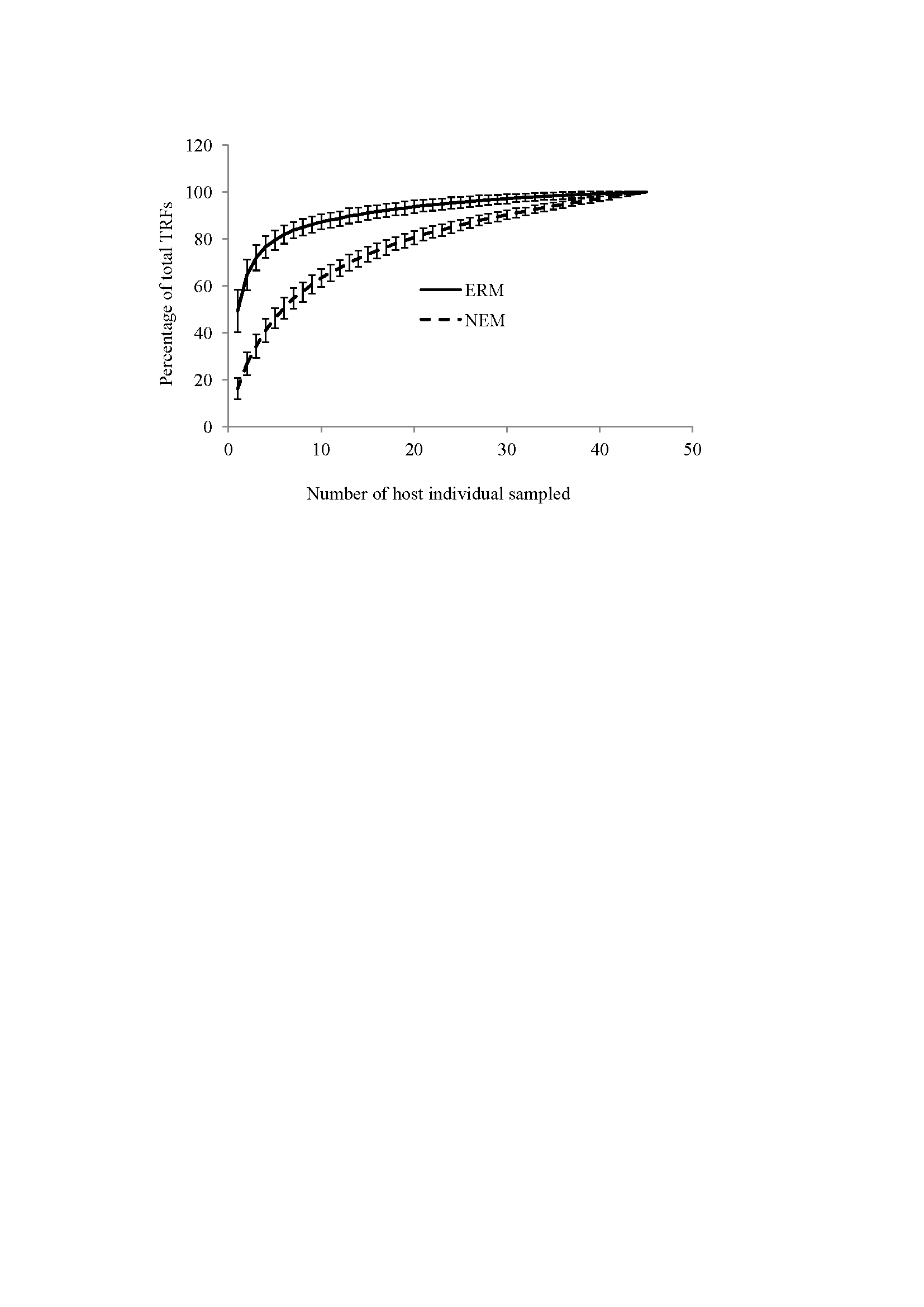

Supplement: Figure S1 — Relationship between percentage of fungal terminal restriction fragments (TRFs) and number of host individuals sampled. Bars were standard deviations (SD). (TIFF) [file pone.0049867.s001.tiff]

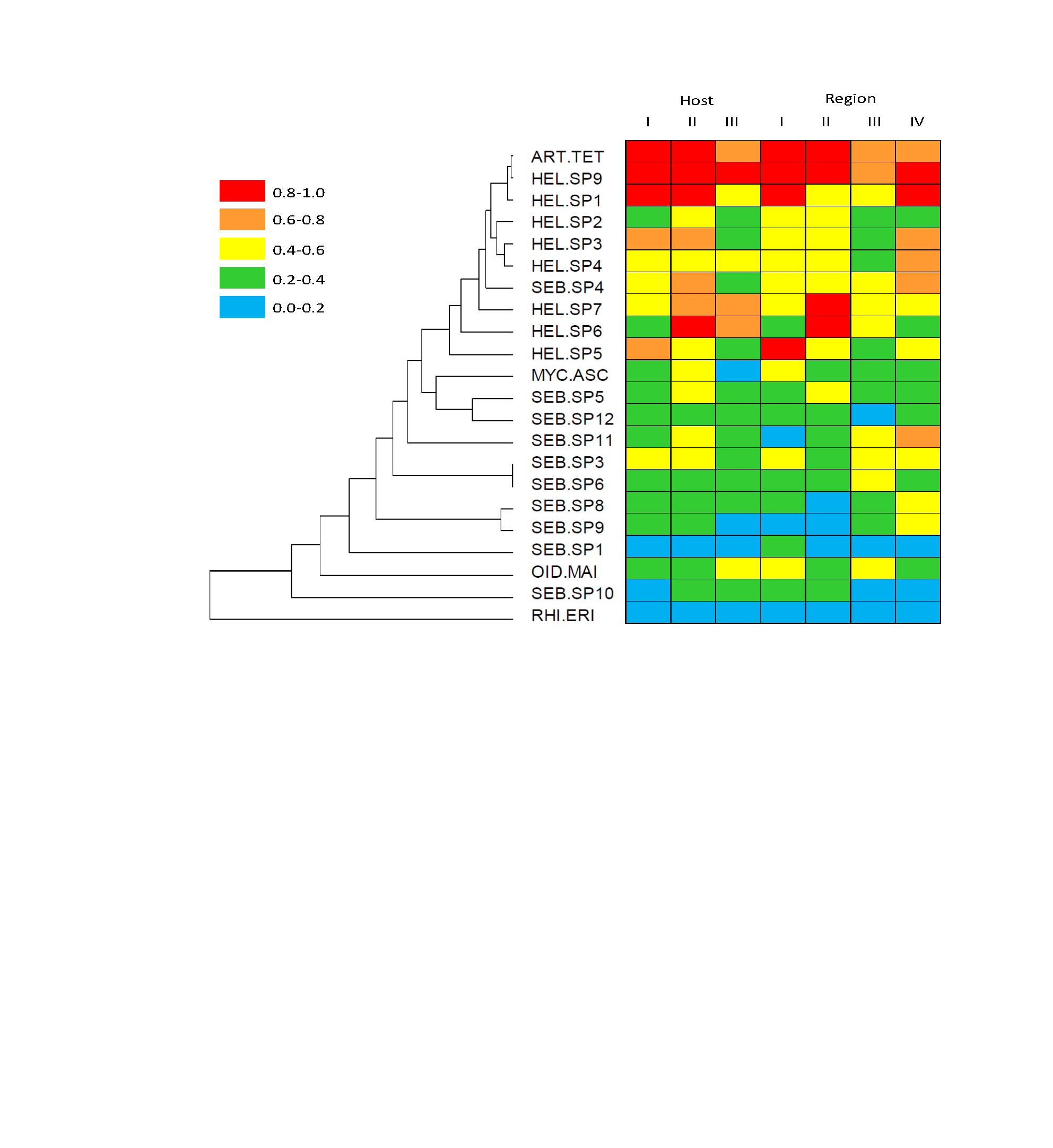

Supplement: Figure S2 — Cluster analysis of distribution patterns of individual fungal species based on Bray-Curtis distance of fungal presence/absence data from 45 sampled plants. The mean relative abundance of each fungal taxon in the three host groups and four sampling regions is shown as a heat-map. (TIFF) [file pone.0049867.s002.tiff]
