# Supplementary material for: Different Distribution Patterns between Putative Ercoid Mycorrhizal and Other Fungal Assemblages in Roots of Rhododendron decorum in the Southwest of China
Source: PLoS One. 2012 Nov 21;7(11):e49867. doi: 10.1371/journal.pone.0049867 (PMC3504031; doi:10.1371/journal.pone.0049867)
Supplement: Table S1 — Climatic, edaphic, and plant element parameters in four sampling regions. Data were shown MAT and MAP are mean annual temperature and mean annual precipitation; STC, STN, and STP indicate soil total carbon, soil total nitrogen and soil total phosphorus; PTC, PTN, and PTP indicate leaf total carbon, leaf total nitrogen and leaf total phosphorus of host plant. Different letters denote significant differences among regions. (DOCX) [file pone.0049867.s003.docx]

**Table S1** Climatic, edaphic, and plant element parameters in four sampling regions. Data were shown MAT and MAP are mean annual temperature and mean annual precipitation; STC, STN, and STP indicate soil total carbon, soil total nitrogen and soil total phosphorus; PTC, PTN, and PTP indicate leaf total carbon, leaf total nitrogen and leaf total phosphorus of host plant. Different letters denote significant differences among regions.

|  | Region I | Region II | Region III | Region IV |
| --- | --- | --- | --- | --- |
| MAT (ºC) | 8.94 ± 0.41c | 10.32 ± 0.33b | 10.19 ± 0.55b | 12.05 ± 0.18a |
| MAP (mm) | 1077 ± 15b | 995 ± 41b | 1212 ± 36a | 1300 ± 32a |
| STC (g/kg) | 55.2 ± 12.1a | 42.2 ± 4.5a | 59.3 ± 12.2a | 65.3 ± 11.7a |
| STN (g/kg) | 4.05 ± 0.67ab | 2.41 ± 0.25b | 3.18 ± 0.65b | 5.38 ± 0.93a |
| STP (g/kg) | 0.98 ± 0.18a | 0.93 ± 0.18a | 1.49 ± 0.33a | 1.18 ± 0.12a |
| PH | 7.15 ± 0.41b | 6.57 ± 0.31b | 6.34 ± 0.45b | 4.72 ± 0.06a |
| PTC (mg/g) | 436.9 ± 6.3a | 410.8 ± 7.3b | 446.1 ± 7.5a | 431.3 ± 3.1a |
| PTN (mg/g) | 13.9 ± 2.6b | 9.0 ± 0.4b | 10.8 ±0.5b | 22.7 ±2.6a |
| PTP (mg/g) | 0.69 ± 0.06b | 1.08 ± 0.11ab | 1.09 ± 0.11ab | 1.59 ± 0.48a |
