# Supplementary material for: Different Distribution Patterns between Putative Ercoid Mycorrhizal and Other Fungal Assemblages in Roots of Rhododendron decorum in the Southwest of China
Source: PLoS One. 2012 Nov 21;7(11):e49867. doi: 10.1371/journal.pone.0049867 (PMC3504031; doi:10.1371/journal.pone.0049867)
Supplement: Table S2 — Sampling sites of Rhododendron decorum. (DOCX) [file pone.0049867.s004.docx]

**Table S2. Sampling sites of *Rhododendron decorum***

| **Region** | **Site** | **Position** | **Altitude (m)** | **Accompanying plants** |
| --- | --- | --- | --- | --- |
| I | S1 | N28°57′50.6″; E102°18′36.2″ | 2 000 | *Rh. yunnanense*,  *Juglans cathayensis*,  *Elaeagnus* sp.,  *Sinarundinaria nitida*,  *Deutzia scabra*. |
|  | S2 | N28°50′41.5″; E102°17′07.1″ | 2 372 | *E.* sp.,  *Alnus cremastogyne*,  *Cotoneaster* sp.,  *Clematis florida*.,  *Cephalotaxus fortunei* |
|  | S3 | N28°33′29.5″;  E102°16′38.7″ | 2 400 | *Rh. irroratum*,  *Rh. spinuliferum*,  *Quercus glandulifera*,  *Schima crenata* |
|  | S15 | N28°37′9.8″; E103°07′8.8″ | 2 304 | *P. densata*,  *Berberis* sp.,  *Co.* sp.,  *Pteridium aquilinum* var. *latiusculum* |
| II | S9 | N27°43′16.4″; E100°55′47.8″ | 2 653 | *P. densata*,  *Q. semicarpifolia*,  *Rh. racemosum* |
|  | S10 | N27°43′03.8″; E100°42′02.7″ | 2 690 | *P. densata*,  *Q. semicarpifolia*,  *Rh. racemosum* |
|  | S11 | N27°10′12.4″; E100°40′44.3″ | 3 039 | *P. densata*,  *Q. semicarpifolia*,  *Rh. racemosum*. |
|  | S12 | N26°53′40.6″; E100°18′53.6″ | 2 677 | *P. densata*,  *Q. semicarpifolia*,  *Rh. racemosum* |
| III | S7 | N27°43′07.7″; E102°21′19.0″ | 2 443 | *E.* sp.,  *A. cremastogyne*,  *Illicium verum*,  *Berberis* sp. |
|  | S8 | N27°32′44.1″; E101°44′14.7″ | 2 907 | *P. densata*,  *Q. semicarpifolia*,  *Lithocarpus* sp.,  *R.* sp. |
|  | S13 | N27°51′23.5″; E100°26′32.6″ | 2 580 | *P. densata*,  *R.* sp.,  *Vaccinium* sp. |
|  | S14 | N27°52′56.0″; E100°32′05.1″ | 3 222 | *P. densata*,  *Q. semicarpifolia*,  *Rh. racemosum* |
| IV | S4 | N26°50′15.4″; E102°14′28.2″ | 2 740 | *A. cremastogyne* |
|  | S5 | N26°49′42.4″; E102°15′7.1″ | 2 543 | *A. cremastogyne* |
|  | S6 | N26°45′19.2″; E102°40′14.1″ | 2 440 | *Pinus armandi*,  *P. densata*,  *Rosa omeiensis*,  *Rh. irroratum*,  *Pyrostegia ignea*. |
