# Supplementary material for: Different Distribution Patterns between Putative Ercoid Mycorrhizal and Other Fungal Assemblages in Roots of Rhododendron decorum in the Southwest of China
Source: PLoS One. 2012 Nov 21;7(11):e49867. doi: 10.1371/journal.pone.0049867 (PMC3504031; doi:10.1371/journal.pone.0049867)
Supplement: Table S3 — Primers used in ISSR analysis for R. decorum. (DOCX) [file pone.0049867.s005.docx]

**Table S3 Primers used in ISSR analysis for *R. decorum***

| Primer | Sequence |
| --- | --- |
| i25 | (AC) _8_T |
| UBC 826 | (AC) _8_C |
| UBC 827 | (AC) _8_ G |
| UBC 835 | (AG) _8_ YC |
| UBC 836 | (AG) _8_ YA |

Note：Y= (C, T)
